# Reliability of the Scapula Reposition Test in Subjects with Rotator Cuff Tendinopathy and Scapular Dyskinesis

**DOI:** 10.3390/jcm9010080

**Published:** 2019-12-28

**Authors:** Ruben Fernandez-Matias, Pablo Gallardo-Zamora, Cristina Lorenzo Sanchez-Aguilera, Hector Mardones-Varela, Tomas Gallego-Izquierdo, Daniel Pecos-Martin

**Affiliations:** 1 Institute of Physical Therapy and Pain, University of Alcala, Ciudad Residencial Universitaria Local 7-8, Alcalá de Henares, 28805 Madrid, Spain; 2 Physiotherapy and Pain Group, Department of Physical Therapy, University of Alcala, Alcalá de Henares, 28871 Madrid, Spain

**Keywords:** shoulder, scapular dyskinesis, rotator cuff tendinopathy, reliability

## Abstract

The Scapula Reposition Test (SRT) is proposed to determine if a relationship exists between scapular dyskinesis and shoulder pathology. The purpose of this study was to evaluate intra-rater and inter-rater reliability of the SRT in subjects with rotator cuff tendinopathy and scapular dyskinesis. In addition, we compared subjective strength findings from the test to an objective measure made by dynamometry. The SRT was independently and randomly performed by two physical therapists in 42 subjects. The percent agreement, Cohen’s kappa (κ), maximum attainable κ, prevalence and bias indexes, and prevalence-adjusted-bias-adjusted kappa were used as intra- and inter-rater reliability estimates. Finally, the point-biserial correlation coefficient (r_pb_) was used for correlation analysis of objective and subjective strength findings. A moderate intra-rater (κ = 0.43; CI 95%, 0.14 to 0.73; *p* = 0.004) and poor inter-rater (κ = 0.08; CI 95%, −0.22 to 0.38; *p* = 0.61) agreement was found. Subjective strength changes during SRT and dynamometry were poorly correlated (r_pb_ = 0.137; CI 95%, −0.175 to 0.423; *p* = 0.389). The SRT cannot be recommended for clinical practice. More studies evaluating its reliability are needed as well as further research on the capability of a rater to manually detect strength changes.

## 1. Introduction

Rotator cuff tendinopathy (RCT), subacromial pain syndrome, and/or rotator cuff related shoulder pain are the most common disorders of the shoulder, accounting for 44%–65% of all complaints of shoulder pain [1]. One factor proposed for the development and maintenance of this disorder is scapular dyskinesis [2]. Scapular dyskinesis is defined as an alteration of the normal position and/or movement of the scapula [3].

There is currently insufficient evidence to support the claim that deviation from a ‘normal’ scapular position and/or movement contributes to RCT [2]. There is also a lack of evidence showing good diagnostic accuracy of scapular dyskinesis orthopedic tests in detecting the presence of shoulder pain [4]. These findings lead to the conclusion that it is not sufficient to diagnose the presence of scapular dyskinesis; rather, one needs to discern when it is involved in patients’ symptomatology [5]. There are two tests proposed in the literature for that intent: The Modified Scapular Assistance Test and the Scapula Reposition Test (SRT) [3].

The aim of the SRT is to detect changes in strength or pain during isometric shoulder abduction with manual correction of the scapula position by inducing it a posterior tilting and external rotation. If there is an increase in shoulder strength or a reduction in shoulder pain, then the test is considered positive, suggesting an implication of scapular dyskinesis in the patient’s disorder [6]. However, despite being recommended for use in clinical practice in the literature, there are currently no studies that have evaluated the reliability of the SRT [7]. Therefore, the main purpose of this study is to examine the reliability of the SRT, which is an indispensable requirement for clinical practice.

## 2. Methods

### 2.1. Study Design

An intra- and inter-rater reliability study was conducted following the recommendations of the Guidelines for Reporting Reliability and Agreement Studies (GRRAS) [8]. Furthermore, correlation between strength-subjective findings from SRT and dynamometry objective measurements were analyzed.

### 2.2. Subjects

A convenience sample of patients was recruited from Hospital Universitario Príncipe de Asturias (Madrid, España) and from announcements in the city. Before participation, all subjects signed a consent form according to the Helsinki Declaration. For the descriptive analysis of the sample, the age, height, weight, gender, symptomatic side, dominant side, pain level in the last week, pain duration and disability information were collected. A visual analogue scale (VAS) was used to rate the pain level in the last week where 0 represents no pain, and 10 represents the worst pain imaginable. VAS has been shown to be a valid and reliable scale [9]. Disability was measured with a Shoulder Pain and Disability Index (SPADI), a valid and reliable questionnaire of which transcultural adaptation from English version to Spanish (Spain) was made in 2015 [10]. Ethical approval was obtained from the Ethical Committee of Hospital Universitario Príncipe de Asturias (OE 17/2018).

According to sample size calculation performed with Epidat 4.2 software, a total of 39 subjects had to be recruited. The sample size calculation was based on detecting a kappa (κ) value of 0.80 with a lower confidence interval (CI) of 0.60 and an upper CI of 1.00. A lower CI value of 0.60 was used following the recommendations from Cadogan [11] and Scholtes [12] on minimal agreement requirements for clinical use of orthopedic tests; 50% positive ratings were assumed for both raters.

### 2.3. Inclusion Criteria

To be included, subjects had to be older than 18 years and present a history of shoulder pain greater than 3 months [13] due to RCT diagnosed with at least 3 out of 5 positive tests: Neer, empty can, painful arc, Hawkins–Kennedy, and external rotation resistance in resting position [14,15]. Patients also had to show scapular dyskinesis according to the Dynamic Scapula Dyskinesis Test [16,17] and be able to actively elevate the arm to 90° and preserve at least 50% of external rotation range of motion compared to the contralateral arm [13].

### 2.4. Exclusion Criteria

Subjects were excluded if they had surgery in last 6 months, suspected any other shoulder pathology, had pain and/or shoulder range of motion that was affected by neck movements, or had systemic diseases or other conditions that interfered with the study [13]. Likewise, analgesic or anti-inflammatory medication use 1 week before measurements was also considered an exclusion criterion.

### 2.5. Raters

Two doctors of physical therapy (DP, CL) with 10 years of clinical experience performed the SRT on patients at the Faculty of Physical Therapy of Alcalá University (Madrid, Spain). Before the study, the raters received an instruction session of two hours on the performance of the SRT. Both examiners performed the test to evaluate inter-rater reliability. One of them (DP) repeated the procedure for intra-rater reliability analysis while the other rater (CL) carried out the dynamometry measurements with the help of a third investigator (RF) in order to be blinded to the results.

## 3. Measurements

### 3.1. Scapula Reposition Test

The SRT was performed at a position of 90° of shoulder elevation in the scapular plane with the thumb pointing up. An elevation angle of 90° was controlled with the use of a digital inclinometer (Baseline 12-1057^®^). The reported intraclass correlation coefficient (ICC) values of the inclinometer are good for both intra-rater (ICC = 0.88) and inter-rater (ICC = 0.92) reliability [18]. Masking tape was applied to the floor at a 30° angle to guide scapular plane position following a similar procedure from a previous study [6].

Raters were located behind and by the side of the shoulder being tested. For scapular repositioning, the palm and the thenar eminence of the hand contacted the spine of the scapula while fingers where placed relaxed over the upper trapezius. The forearm was placed in the medial border of the scapula toward the inferior angle. Palpation of the above-mentioned bone references is valid and reliable [19]. On the other hand, to evaluate upper limb strength, the last four fingers were placed just proximal to the styloid radius apophysis.

Subjects were asked to hold a 5 s maximum isometric contraction first without (S1) and then with (S2) scapula repositioning. There was a 30 s rest interval between S1 and S2 and a 5 min rest between different raters. Patients also had to rate their pain level on a VAS during isometric contraction in both situations.

Both raters were blinded to each other’s findings and to the patients’ self-rated pain level. Furthermore, the raters had no access to clinical information such as grade of disability, pain intensity, and other data related to the subjects being tested. The Epidat 4.2 software randomized the rating order of the examiners.

Despite the resting time given, repeated measures may have influenced patient’s symptoms. To avoid this, the pain level at rest was rated before each SRT performance according to the recommendations of Kopkow [20]. The same measurement process was repeated on a consecutive day by one of the raters to report the intra-rater reliability. Due to the nature of the SRT, it was impossible to blind this rater to his own previous strength-subjective findings.

### 3.2. Dynamometry

An objective strength measure was performed on the second day with the use of a handheld dynamometer (microFET 2^TM^ model, Hoggan Health Industries Inc., West Jordan, UT, USA). Subjects rated the pain intensity level at rest before each test. Both testing situations, S1 and S2, were repeated three times with a 30 s rest interval between each measurement. Also, two minutes were left between different situations. The process was then repeated after a 5 min rest. Epidat 4.2 software randomized the order of the situation tested first. The mean of the three measures was included in the statistical analysis. In order to blind the rater who performed the reposition of the scapula during the dynamometry, another investigator (RF) recorded the findings. The rater was also blinded to the pain level of the subject.

**Figure 1:**
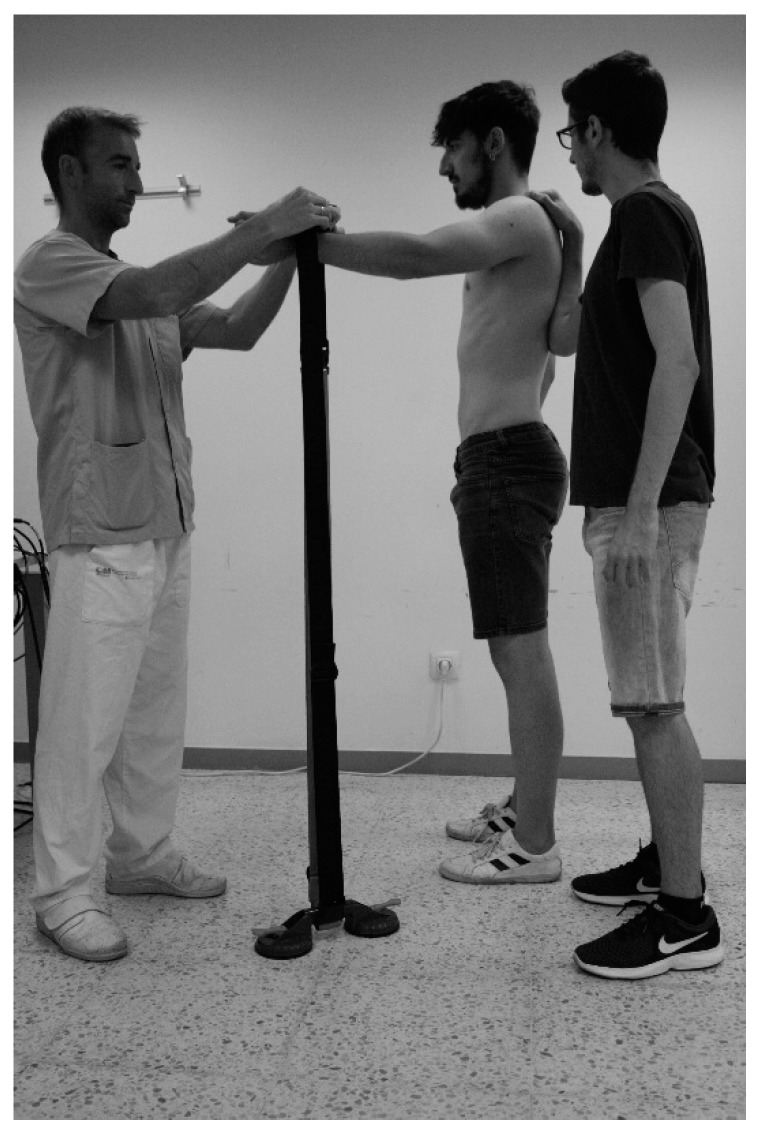
Attachment system for dynamometer measurements.

Good intra-rater and inter-rater agreement has been reported when using a dynamometer in healthy (ICC = 0.93, ICC = 0.83, respectively) and symptomatic people (ICC = 0.96, ICC = 0.97, respectively) [21]. The influence of the rater during dynamometry is a potential source of error so an attachment system based on a strap and a suction pad stuck to the floor was used (Figure 1).

### 3.3. Statistical Analysis

Percent agreement, Cohen’s Kappa (κ), maximum attainable κ (κ_max_), prevalence index (PI), bias index (BI), and prevalence-adjusted-bias-adjusted kappa (PABAK) values with 95% confidence interval (CI) were used as estimates of intra- and inter-rater reliability of the SRT [22]. The K values were interpreted following the recommendation of Landis and Koch [23].

The change in pain during the SRT was measured with VAS and was dichotomized using a cut-off point of 2 cm [9,24]. If there was a reduction in pain greater than 2 cm, then the test was considered positive for pain criteria. The overall SRT was considered positive if there was a positive result in pain or strength criteria and negative if both were negative.

An intra-test reliability analysis of shoulder abduction dynamometry with and without scapula repositioning was also conducted. The ICC was calculated under the assumption of a two-way mixed model with absolute agreement and average score ICC (3,3) with a 95% CI [25]. The standard error of measurement (SEM) was determined using the formula [26]:

(1) $$SD\times \sqrt{\left(1-ICC\right)}$$

The minimal detectable change with 90% confidence bounds (MDC90) was determined using the formula [26]:

(2) $$SEM\times \sqrt{2}\times 1.64$$

SEM and MDC90 were also reported as a percentage of the sample mean. Bland–Altman plots were also constructed [26].

The relationship between subjective strength changes detected by the therapist during SRT and dynamometry was analyzed with the point-biserial correlation coefficient (r_pb_) [27]. Pairwise comparisons of the pain level at rest before performance of each test procedure were performed with Student’s *t*-test using the Bonferroni adjustment method [27].

Kappa analyses were performed with statistical software R Version 3.5.3 (R Core Team (2019). R: A language and environment for statistical computing (R Foundation for Statistical Computing, Vienna, Austria). All other analyses were performed with SPSS V.22 (SPSS Inc., Chicago, IL, USA). All analyses were conducted considering an αlevel of 0.05.

## 4. Results

**Figure 2:**
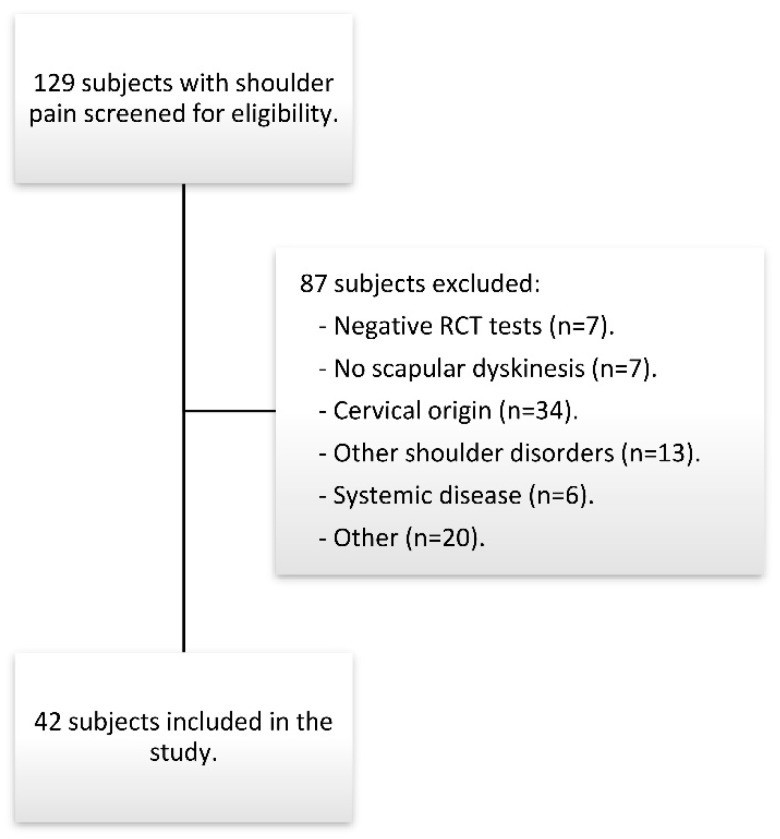
Flow diagram of subjects.

The final sample was composed of 42 subjects (Figure 2). The characteristics of the patients are presented in Table 1.

### 4.1. Pairwise Comparisons of Pain Ratings at Rest

Statistically significant differences were found between pain rating at rest before the first and the second dynamometry (mean difference = 0.57; 95% CI, 0.17 to 0.97; *p* = 0.001). None of the other pairwise comparisons were significant.

### 4.2. Reliability of the SRT

The results of the intra-rater and inter-rater agreement are presented in the two by two contingency tables (Table 2 and Table 3). The intra-rater agreement was moderate (κ = 0.43; 95% CI, 0.14 to 0.73; *p* = 0.004) and the inter-rater agreement was slight (κ = 0.08; 95% CI, −0.22 to 0.38; *p* = 0.61). The PABAK value for intra-rater agreement was 0.57 (95% CI, 0.26 to 0.79) and 0.19 (95% CI, −0.13 to 0.49) for inter-rater agreement. Percent agreement, PI, BI, and κ_max_ values are presented in Table 4.

### 4.3. Dynamometry Reliability

**Figure 3:**
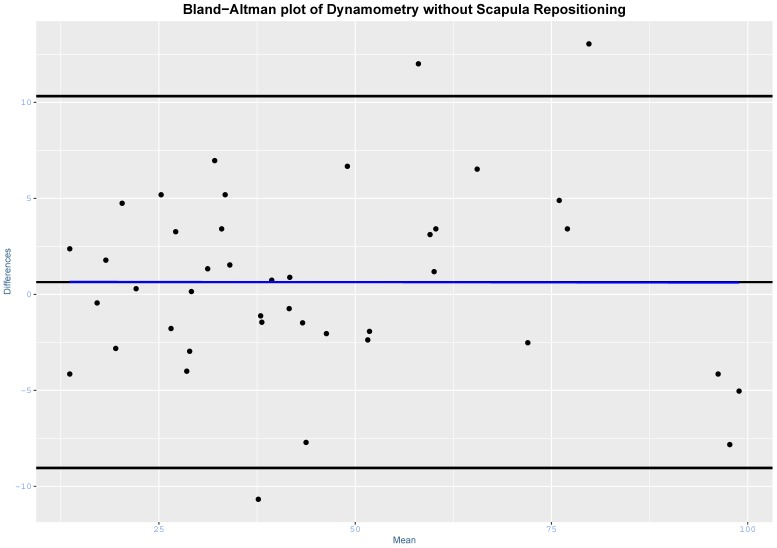
Bland–Altman plot for dynamometry without scapula repositioning. Blue line = regression line.

**Figure 4:**
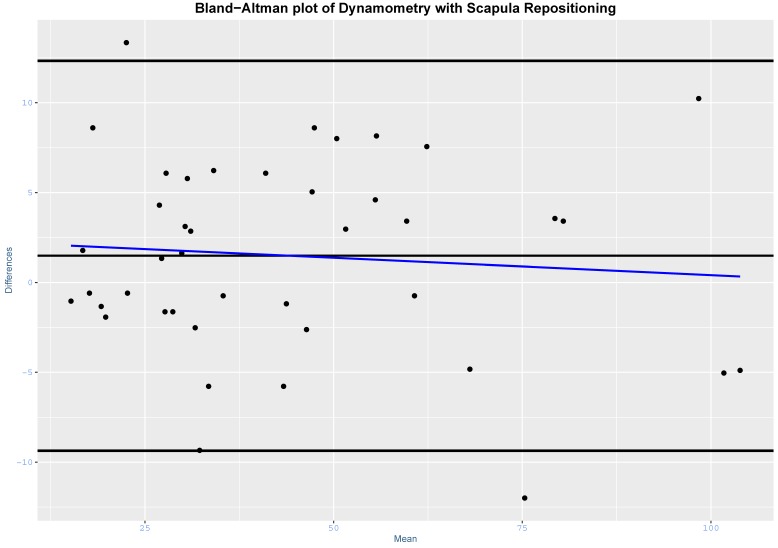
Bland–Altman plot for dynamometry with scapula repositioning. Blue line = regression line.

The intra-test reliability was high with scapula repositioning (ICC_3,3_ = 0.986; 95% CI, 0.974 to 0.993; SEM = 4.00; %SEM = 8.96; MDC90 = 9.28; %MDC90 = 20.78) and without scapula repositioning (ICC_3,3_ = 0.990; 95% CI, 0.981 to 0.994; SEM = 3.39; %SEM = 7.60; MDC90 = 7.86; %MDC90 = 17.62). Bland–Altman plots are presented in Figure 3 and Figure 4.

### 4.4. Correlation Analysis

There was a poor correlation between subjective-strength changes detected by the therapist during SRT and dynamometry measurements (r_pb_ = 0.137; 95% CI, −0.175 to 0.423; *p* = 0.389).

## 5. Discussion

### 5.1. Scapula Reposition Test Reliability

Despite SRT use recommendations in the literature, its reliability had not yet been tested [7]. A moderate intra-rater (κ = 0.43) and a poor inter-rater (κ = 0.08) reliability were obtained here. Neither result exceeds the lower limit of 0.60 proposed by Cadogan [11] and Scholtes [12] as the minimal agreement value required for clinical use of an orthopedic test. Furthermore, the PABAK values of 0.57 and 0.17 suggest that SRT is not a reliable test. κ values have differences between intra- and inter-rater reliability and may be due to the impossibility of blinding the rater to his own previous strength evaluation.

Raters were experienced in the treatment of patients with RCT but not in the performance of SRT. Despite the accomplishment of the training session, several authors consider that the amount of experience in the test procedure may affect the results [8,28,29]. The findings can be extrapolated to equally experienced professionals with more confidence. However, experience was likely not a decisive factor on our results due to the difficulties in determining when the rater is sufficiently trained in a test procedure, the simplicity of SRT, and the clinical experience of both raters.

The subjects did not perform any practice before the measurement process. Tate et al. [6] had recommendations on this topic based on a Kibler et al. [30] study which found an increase in strength in all subjects after scapula reposition. In Kibler’s study, the test was always performed without repositioning in the first place, and this might explain those findings. In our study, patients were only given a visual and verbal explanation of the test before the performance. Despite the fact we did not obtain similar results to Kibler et al. [30], we cannot guarantee that the lack of prior training did not affect the results.

This test was initially described by Kibler as the Scapula Retraction Test and was performed with the shoulder in an empty can position. This position presumably increases the supraspinatus activation [30]. However, the empty can position does not isolate supraspinatus activation more than a full can position does, as a later study reported [31]. Thus, the full can position was chosen, as it was considered more comfortable to the patients.

It is important to emphasize the homogeneity of the cohort and the presence of scapular dyskinesis in all subjects. Rabin et al. [32] and Kopkow et al. [20] studied the reliability of the Scapular Assistance Test, but they did not select patients with the same shoulder pathology, and scapular dyskinesis was not considered as an inclusion criteria. This surely influenced the interpretation of the findings.

### 5.2. Manual Test and Dynamometry Correlation

A statistically significant difference was found in pain intensity levels before the two dynamometry measurements (mean difference = 0.569). However, it was not considered clinically relevant because only four subjects exceeded the minimum detectable change of two centimeters in the VAS [9,24] with a lower value of 2.21 and an upper of 2.53. The subjects were not considered to have an impact on dynamometry reliability, so they were not excluded from analysis. High reliability rates were obtained in both S1 (ICC_3,3_ = 0.990) and S2 (ICC_3,3_ = 0.986) similar to Tate et al. [6] (0.982 and 0.964, respectively).

Poor correlation was found between the raters’ subjective strength criteria during the SRT and dynamometry (r_pb_ = 0.137). This highlights the limited capability of manual muscle testing to detect changes in shoulder abduction strength. In a previous study, Nagatomi et al. [33] obtained a 94.3% diagnostic accuracy in detecting a change in shoulder abduction strength for a 21.1% cut-off point difference when the contralateral side was considered. However, different shoulder pathologies from that of the present study were also analyzed. Nagatomi et al. [33] also tested both upper limbs simultaneously. Assessing the strength from both upper limbs at the same time might be more accurate than evaluating it over two different moments. Therefore, the poor correlation observed here questions the validity of the SRT strength criteria to decide whether scapular dyskinesis plays a role in a patient’s symptoms.

## 6. Conclusions

In summary, our results cannot recommend the use of the SRT in clinical practice. Thus, a scapular dyskinesis modification approach built on a SRT outcome is not justified. However, further research on the capability of manual tests to detect changes in strength are needed, as are more studies investigating the SRT reliability in other samples and with other raters.

## Figures and Tables

**Table 1:** Patient characteristics (*n* = 42).

Characteristic	Average (SD)
Age, years	53.55 (12.15)
Height, cm	166.86 (9.67)
Weight, kg	74.96 (17.10)
Body mass index	26.17 (5.09)
Sex, *n* (%)	
Female	21 (50)
Male	21 (50)
Tested side, *n* (%)	
Right	18 (42.90)
Left	24 (57.10)
Dominant side, *n* (%)	
Right	42 (100)
Left	0 (0)
VAS pain, cm	4.07 (1.80)
Pain duration, months	22.83 (24.03)
SPADI, %	43.88 (16.64)

SD = standard deviation, cm = centimeters, kg = kilograms, VAS = Visual Analogue Scale, SPADI = Shoulder Pain and Disability Index.

**Table 2:** Two by two contingency table for intra-rater agreement.

		Rater 1 Second Trial		Total
		Negative	Positive	
**Rater 1 First Trial**	**Negative**	6	6	12
	**Positive**	3	27	30
**Total**		9	32	42

**Table 3:** Two by two contingency table for inter-rater agreement.

		Rater 2		Total
		Negative	Positive	
**Rater 1 First trial**	**Negative**	5	7	12
	**Positive**	10	20	30
**Total**		15	27	42

**Table 4:** Intra- and inter-rater reliability of the Scapula Reposition Test.

	Observed Agreement (%)	Cohen’s κ (95% CI)	Prevalence Index (95% CI)	Bias Index (95% CI)	PABAK (95% CI)	κ_max_ (95% CI)
**Intra-rater**	33/42 (78.57)	0.43 (0.14, 0.73)	−0.50 (−0.68, −0.32)	0.07 (−0.11, 0.26)	0.57 (0.26, 0.79)	0.81 (0.60, 1.00)
**Inter-rater**	25/42 (59.52)	0.08 (−0.22, 0.38)	−0.36 (−0.54, −0.18)	−0.07 (−0.27, 0.13)	0.19 (−0.13, 0.49)	0.84 (0.66, 1.00)

κ = Kappa, CI = confidence interval, PABAK = prevalence-adjusted-bias-adjusted-kappa, κ_max_ = maximum attainable kappa.
